# Musical Tension Associated With Violations of Hierarchical Structure

**DOI:** 10.3389/fnhum.2020.578112

**Published:** 2020-09-18

**Authors:** Lijun Sun, Li Hu, Guiqin Ren, Yufang Yang

**Affiliations:** ^1^Key Laboratory of Behavioral Science, Institute of Psychology, Chinese Academy of Sciences, Beijing, China; ^2^Department of Psychology, University of Chinese Academy of Sciences, Beijing, China; ^3^College of Psychology, Liaoning Normal University, Dalian, China

**Keywords:** musical tension, hierarchical structure, global field power, N5, alpha

## Abstract

Tension is one of the core principles of emotion evoked by music, linking objective musical events and subjective experience. The present study used continuous behavioral rating and electroencephalography (EEG) to investigate the dynamic process of tension generation and its underlying neurocognitive mechanisms; specifically, tension induced by structural violations at different music hierarchical levels. In the experiment, twenty-four musicians were required to rate felt tension continuously in real-time, while listening to music sequences with either well-formed structure, phrase violations, or period violations. The behavioral data showed that structural violations gave rise to increasing and accumulating tension experience as the music unfolded; tension was increased dramatically by structural violations. Correspondingly, structural violations elicited N5 at GFP peaks, and induced decreasing neural oscillations power in the alpha frequency band (8–13 Hz). Furthermore, compared to phrase violations, period violations elicited larger N5 and induced a longer-lasting decrease of power in the alpha band, suggesting a hierarchical manner of musical processing. These results demonstrate the important role of musical structure in the generation of the experience of tension, providing support to the dynamic view of musical emotion and the hierarchical manner of tension processing.

## Introduction

Music is universal and essential in human societies, owing to its strong power of emotion (e.g., Koelsch, 2014; Swaminathan and Schellenberg, 2015). However, it remains unclear how the auditory elements in music generate emotion responses (Juslin and Laukka, 2004; Juslin and Västfjäll, 2008; Juslin, 2013). In the literature of music theories and music psychology, tension is considered as the link between musical events and experienced emotions (Lehne et al., 2013; Lehne and Koelsch, 2015b) and so that as an appropriate point of penetration to investigate the dynamics and richness of musical emotion. Musical tension is an affective state that is associated with conflict, dissonance, instability, or uncertainty and create a yearning for resolution (Lehne and Koelsch, 2015b). It is believed that the recurrent alterations between tension and relaxation create a music-generated experience (Lerdahl and Jackendoff, 1983).

As to the relationship between musical structure and tension, presently most studies have primarily focused on the relationship between tonally hierarchical structure and tension, and found that tonal regularity plays a critical role in tension generation; moving away from tonal center would induce tension, whereas returning to the tonal center a sense of resolution (Bigand et al., 1996; Bigand and Parncutt, 1999; Steinbeis et al., 2006; Lerdahl and Krumhansl, 2007). For example, Bigand and his colleagues manipulated the stability of certain chords in music sequences and examined the tension rating of the key chords, suggesting that unstable chords gave rise to higher tension experience than stable chords (Bigand et al., 1996; Bigand and Parncutt, 1999). Steinbeis et al. (2006) constructed harmonic regular and irregular conditions by manipulating the last chord of short and real music pieces. The results showed that structural violations induced a strong sense of tension because the ending of the music did not fulfill the expectation. While these studies shed light on the relationship between tonal structure and the experience of tension, musical stimuli used in these studies were short of large-scale structures.

According to the generative theory of tonal music (GTTM) and tonal tension model (TTM), music expresses complicated meaning and emotion with hierarchical structures. Not only each musical event expresses specific tension and relaxation, but the events are also organized into a hierarchy with a prolongational reduction tree, creating a complex pattern of tension and resolution (Lerdahl and Jackendoff, 1983; Lerdahl and Krumhansl, 2007). In real music pieces, discrete elements are organized at multiple time scales to form structural units such as musical section, phrase, period and movement (Lerdahl and Jackendoff, 1983). Based on different hierarchical structural units, multiple local patterns of tension and relaxation constitute a global pattern of tension and relaxation. That is, small tension arches are always embedded in large tension arches based on the hierarchical structure with different timescales in music. Based on the structural relations in music, several tension arches overlap and interweave to develop the tension and resolution in large-scale structures (Koelsch, 2012).

Music psychology has explored the cognitive and neural bases of tension generation. The model of music tension proposed by Margulis (2005) suggests that listeners make predictions for the upcoming events continuously based on the musical context. The mismatch between the actual stimulus and the prediction in the brain will induce tension experience, such as tonal inconsistency and violations. Recently, a general model of tension and suspense has been proposed by Lehne and Koelsch (2015a) based on the predictive coding theory (Friston and Kiebel, 2009). They suggest that prediction errors enable the brain to update predictions for musical events in the process of music listening. Specifically, when an unpredicted event occurs in music progression, listeners’ mental models are not maintained stable, but rather updated based on the current perceptual information. In other words, prediction violations in music could lead to predictive errors and event model resetting, which dynamically engaged working memory and cognitive control processes, such as attention switching (Zacks et al., 2007; Kurby and Zacks, 2008).

In terms of the proposals as to cognitive bases underlying tension generation, we assumed that musical tension could be modulated by the hierarchical levels of musical structure. That is, compared with low-level units, the processing of high-level units would be more difficult and required more cognitive efforts. The difficulties may result from: first, much more information included in musical events at higher levels; second, long-distance dependency involved in integrating multiple small units into a large unit at higher levels. The evidence has been provided by previous studies. It was found that larger closure positive shift (CPS) was elicited by period boundaries than section and phrase boundaries in music, suggesting more retrospective processing of preceding information (Zhang et al., 2016); it was more difficult for a global structure to be integrated into the context than the local structure (Zhang et al., 2018).

How tension experience is modulated by hierarchical structures with different time scales? Whether higher tension would be induced by structural violations at a higher level than that at a lower level? What are the cognitive neural bases underlying the music tension generation? Using continuous behavioral rating and electroencephalography (EEG), the present study explored these questions by investigating the processing of tension experience and neural responses induced by structural violations at different hierarchical levels. In the experiment, four-phrase music sequences were used to organize hierarchical units at different timescales. There were three types of music structures, in which the consistency of the tonality at different hierarchical levels was manipulated, while the musical structure within each phrase was kept the same across conditions. In well-formed sequences, four phrases were organized into a structure with three hierarchical levels and had consistent tonality from the beginning to the end. In sequences with phrase violations and period violations, the tonal consistency was disrupted at either phrase or period boundary respectively.

We hypothesized that tension ratings could be raised by structural violations, and higher tension would be induced by higher-level violations than the lower ones. Furthermore, for the cognitive mechanism underlying tension experience, larger N5 response, a neurophysiological marker of the integrated process of musical structure, would be elicited by sequences with structural violations compared to the well-formed sequences (Poulin-Charronnat et al., 2006; Loui et al., 2007; Miranda and Ullman, 2007; Koelsch and Jentschke, 2010; Sun et al., 2020b). Also, we predicted that the power in the alpha frequency band would decrease in response to structural violations compared with the well-formed sequences since the alpha power was tightly associated with the cognitive resources and attention (Meyer et al., 2013; Sadaghiani and Kleinschmidt, 2016). Finally, we hypothesized that both the N5 component and the power of alpha-band would be influenced by the hierarchical level of structural violations because more cognitive resources were required for working memory updating and attention switching in large structural units than the small ones.

## Materials and Methods

### Participants

Twenty-four musicians, highly proficient in Western tonal music (*M*_age_ = 23.21 years, *SD* = 2.38, 17 female) participated in the experiment. They had received formal Western instrumental training for an average of 15 years (ranging from 8 to 22 years), playing piano, violin, viola, cello, or tuba. They were all right-handed with normal hearing. None of them reported a history of neural impairment or psychiatric illness. The study was approved by the Institutional Review Board of the Institute of Psychology, Chinese Academy of Sciences, and accords with the ethical principles of the Declaration of Helsinki. All participants were provided with informed consent.

### Stimuli

**Figure 1:**
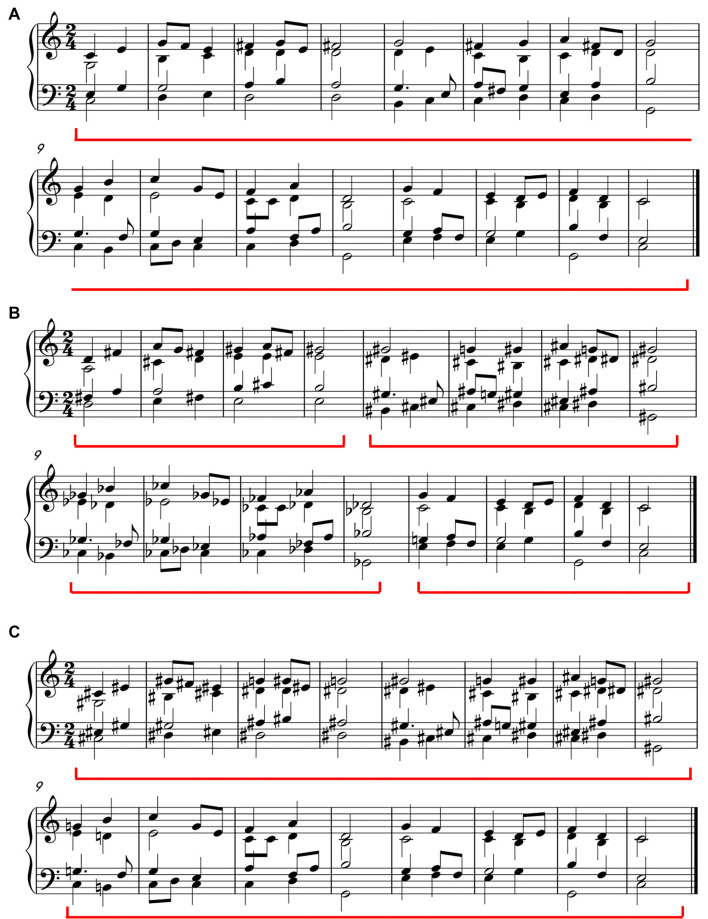
Samples of the musical sequences used in the study. Condition 1: a sequence with a well-formed hierarchical structure **(A)**. Condition 2: a sequence with phrase violations **(B)**. Condition 3: a sequence with a period violation **(C)**.

Ten original chorale sequences were composed in 2/4 meter. Four phrases were organized into a well-formed structure with multi-level hierarchies in each original sequence. As illustrated in Figure 1A (condition 1), the first two phrases and the last two phrases grouped into two music periods with a consistent tonality relationship. There were no pauses during the whole musical sequence. Musical units were mainly separated by the different durations of notes. The boundaries of phrase and period were signified by half notes, which lasted for 1,200 ms and were longer than other notes. Based on each original sequence, two modified versions (condition 2 and condition 3) were created (see Figures 1B,C). The modified versions had structural violations at phrase or period level, while the local structure within each phrase was consistent across conditions. In condition 2, the musical structure was violated at the phrase level by modifying the tonality consistency of each phrase. Thus, the expectation of the listeners cannot be fulfilled at the beginning of each phrase. In condition 3, the musical structure was violated at the period level by modifying the tonality consistency of the two periods. Under such a situation, the expectation of the listeners cannot be fulfilled at the beginning of the second period (that is also the beginning of the third phrase).

The 30 chorale sequences (10 sequences for each condition) were then transposed to three other keys to form a total of 120 sequences. In terms of the sequences, all stimuli were created using the Sibelius 7.5 software (Avid Tech. Incorporated) and were exported in mid format. Using Cubase 5.1 software, the mid files were set at a constant velocity of 100 and were archived with a Yamaha piano timbre at a tempo of 100 beats per minute in.wav format.

### Procedure

There were three versions of sequences: original version with no violations, a modified version with violations at the phrase level, another modified version with violations at the period level. Each version has 40 sequences, resulting in 120 chorale sequences in total. They were presented in a pseudorandom order under two constraints: a given version could not be repeated more than three times in succession, and consecutive sequences were not to originate from the same original chorale sequence.

To reveal the dynamic qualities of the tension experience, participants were required to continuously make real-time judgments of the felt tension using the computer mouse. Tension values were recorded using the Psychpy 1.0. software interface and data were collected at a sampling rate of 20 Hz. Before the beginning of each trial, they will see a sliding bar, raging from 0 to 100, located at the center of the screen. The task for participants was to move the slider up or down through the mouse to indicate the degree of tension they felt while listening to music. Consistent with previous studies (Steinbeis et al., 2006; Lehne et al., 2013), the meaning of tension was not defined by specific descriptions. The initial position of the slider was set at 25 to prevent the rating bar from reaching maximal values throughout the musical progression. The experiment was conducted in an acoustically and electrically shielded room. All stimuli were presented binaurally through Audio Technica CKR30iS headphones. The loudness was adjusted by participants to their comfort levels. Three practice trials were performed before the experimental session to familiarize participants with the stimuli and procedure.

### EEG Recording and Analysis

Using Brain Products (Munich, Germany), EEG data were recorded from 64 Ag/AgCI electrodes mounted at International 10–20 system scalp locations. The data were digitized at a rate of 500 Hz with an additional notched filter at 50 Hz. The FCz was used as an online reference electrode, and an electrode placed between Fz and FPz served as the ground electrode. Blinks and vertical eye movements were recorded with an electrode below the right eye. The impedance of all electrodes was maintained less than 5 kΩ and EEG data were amplified with AC amplifiers.

The raw EEG data were preprocessed with EEGLAB (Delorme and Makeig, 2004) in a MATLAB environment. The continuous data were referenced offline to the algebraic mean of the left and right mastoid electrodes. For the ERPs analysis, the data were filtered offline with a band-pass filter of 1–30 Hz. The high-pass filter of 1 Hz was applied to be consistent with previous literature and assure methodological comparison with other studies (e.g., Hu et al., 2014; Kuhn et al., 2015). Then, the data were segmented into epochs of 22.2 s ranging from 1,000 ms before the onset of the first chord, to 2 s after the offset of each chorale sequence. Next, trials were baseline corrected using the 1,000 ms pre-stimulus interval. The data of each participant were then corrected using an Independent Component Analysis (ICA) algorithm (Makeig et al., 1997; Delorme and Makeig, 2004) implemented in EEGLAB to delete ocular and muscle activity-generated artifacts. After the procedure of ICA components removal, we inspected epochs for each participant visually and rejected the contaminated epochs. Epochs in the same experimental condition were averaged for each participant, yielding three average waveforms in each participant.

Single-subject average waveforms were subsequently calculated to obtain global field power (GFP) values at each instant. At any instant, GFP is calculated as the root of the mean of the squared potential differences between each electrode and the mean of the instantaneous potential across electrodes (Lehmann and Skrandies, 1980). To determine whether the GFP values elicited by the three conditions differed as a function of musical hierarchical structure, we performed a point-by-point repeated measures analysis of variance (RM ANOVA) test. After the significance level (*p*-value) was corrected for using a false discovery rate (FDR) procedure (Durka et al., 2004), a time course of *p* values was obtained. In searching for the time window corresponding to *p* values below 0.05, we decided on the latency of the EEG responses elicited by the three conditions. Then, within the time window identified as being statistically significant, overall ANOVA tests were followed up with further paired comparisons. Finally, given that the strongest filed potentials and highest topographic signal-to-noise ratios were represented by local maxima of the GFP curve (Lehmann and Skrandies, 1980), we calculated scalp topography around 20 ms, on average, from the GFP peaks and compared differences among the three conditions.

For the time-frequency analysis, the preprocessing steps remained the same as for the ERP analysis, other than filtering with a 1–100 Hz band-pass filter. To obtain time-frequency distributions, Fast Fourier Transforms (FFTs) with a fixed 600 ms Hanning window was applied to each epoch for each participant using time steps of 4 ms and frequency steps of 1 Hz. The time-frequency distributions were corrected with a baseline of an interval from 800 to 200 ms pre-stimulus to include subtle stimulus-induced changes in ongoing oscillatory power. Based on previous findings (Knyazev et al., 2008; Parvaz et al., 2012), we selected alpha band frequencies (8–13 Hz) and nine central-frontal electrodes including Fz, F1, F2, FCz, Fc1, Fc2, C1, Cz, C2 electrodes as the regions of interest (ROIs). A point-by-point RM ANOVA test was performed and the significance level (*p*-value) was corrected using a FDR procedure (Durka et al., 2004). The time window of statistical significance was defined as that in which the *p* values were below 0.05. Next, further paired comparisons were individually performed within each significant time window.

## Results

### Behavioral Results

**Figure 2:**
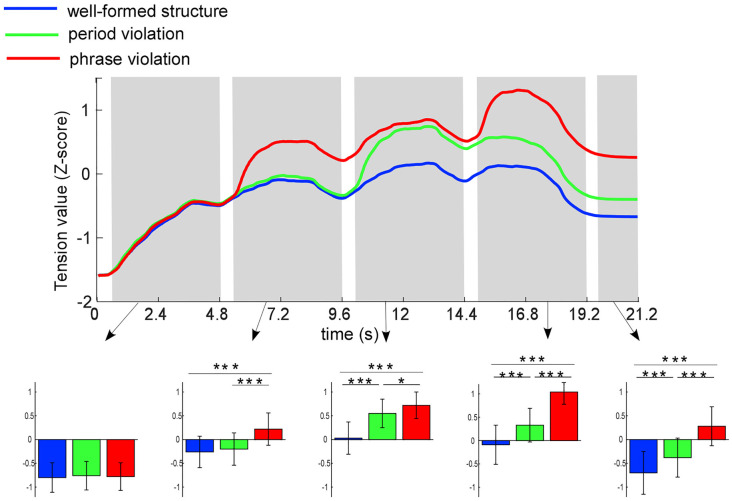
Mean ratings (*z*-score) of tension values for the three conditions at each time point. Five time-windows are analyzed: 0.5–4.8, 5.3–9.6, 10.1–14.4, 14.9–19.2, and 19.7–21.2 s, which marked in gray bars. Colored bars represent the mean of the tension values for the three conditions (expressed as mean ± SD). *p < 0.05; ***p <0.001.

To minimize the differences in slider moving ranges across participants, data were normalized to a zero mean and unit standard deviation (*z*-score) for each participant. Tension ratings were averaged across participants under each condition at each time point. Figure 2 shows average tension profiles under the three conditions, indicating dynamic changes in tension throughout the whole musical pieces. In all conditions, within each phrase, tension values always increased from the beginning to the middle, then declined until the end. Across all three conditions, tension values overlapped only in the first phrase and separated in the other three.

The tension values were averaged for each participant in the following five time-windows: 0.5–4.8, 5.3–9.6, 10.1–14.4, 14.9–19.2, and 19.7–21.2 s (corresponding to the time windows of phrases 1–4 and 1.5 s after the end of sequences). In order to reduce the influence of the response delay, data in the time windows of 0–500 ms after phrase onsets were not analyzed. Two-way RM ANOVA taking time window and condition as within-subject factors was conducted. The results showed that the interaction effect between condition and time window (*F*_(8,184)_ = 30.07; *p* < 0.001, partial *η*^2^ = 0.57), and the main effects of condition (*F*_(2,46)_ = 46.70; *p* < 0.001, partial *η*^2^ = 0.67) and time window (*F*_(4,92)_ = 33.41; *p* < 0.001, partial *η*^2^ = 0.59) were significant. Then, we conducted ANOVA test taking condition as within-subject factor in each time window. The results revealed significant main effects for all time windows except for the first phrase (the first phrase: *p* = 0.08; the second phrase: *F*_(2,46)_ = 32.22; *p* < 0.001, partial *η*^2^ = 0.58; the third phrase: *F*_(2,46)_ = 42.76; *p* < 0.001, partial *η*^2^ = 0.65; the last phrase: *F*_(2,46)_ = 45.62; *p* < 0.001, partial *η*^2^ = 0.67; post-ending: *F*_(2,46)_ = 38.84; *p* < 0.001, partial *η*^2^ = 0.63).

Further paired comparisons among the three conditions showed that for the second phrase, more tension was induced by phrase violations than well-formed structures (*p* < 0.001) and period violations (*p* < 0.001); however, no significant difference of tension was found in conditions between well-formed structures and period violations (*p* = 0.24). For the third phrase, more tension was induced by phrase violations and period violations than well-formed structures (*p*s < 0.001), and more tension was induced by period violations than phrase violations (*p* = 0.049). For the last phrase and post-ending, more tension was induced by phrase violations than in the other two conditions (*p*s < 0.001), while more tension was induced by period violations than well-formed structures (*p*s < 0.001).

### ERP Results

**Figure 3:**
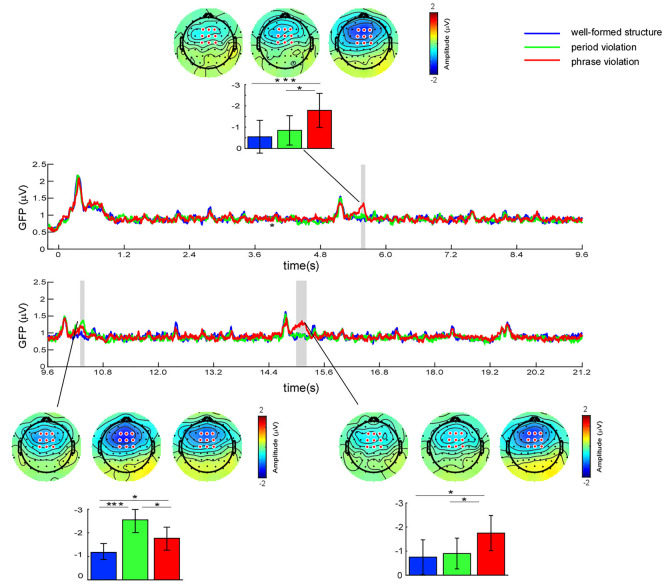
Global field power (GFP) profiles for the three conditions over the whole musical sequences. Significant regions are marked in the gray bar [*p* < 0.05, one-way analysis of variance (ANOVA) test, and false discovery rate (FDR)-corrected]. The scalp topographies were obtained around the GFP peaks for different conditions within the time window of 5,532–5,572 ms (732–772 ms after the onset of the second phrase), 10,350–10,390 ms (750–790 ms after the onset of the third phrase), and 15,132–15,172 ms (732–772 ms after the onset of the last phrase). Colored bars represent the mean of the GFP values and the amplitude of the N5 component at central-frontal electrodes for the three conditions (expressed as mean ± SD). *p < 0.05; ***p < 0.001.

GFP profiles and scalp topography at the instants corresponding to the GFP peaks under different conditions are shown in Figure 3. The segregation of GFP profiles under the three conditions is shown around 700 ms after the onsets of two to four phrases.

Point-by-point RM ANOVA test found that the difference across conditions was significant in the time windows of 5,544–5,590 ms (744–790 ms after the onset of the second phrase), 10,342–10,396 ms (742–796 ms after the onset of the third phrase), and 14,960–15,204 ms (560–804 ms after the onset of the last phrase). Given that frontal-distributed negativities were elicited at the instants corresponding to GFP peaks, further paired comparisons were performed using the average amplitudes at frontal electrodes within the time window of 40 ms around each GFP peak. We conducted a one-way ANOVA taking condition as a within-subject factor in each time window. The results revealed significant main effects for all time windows (5,532–5,572 ms: *F*_(2,46)_ = 7.03; *p* = 0.002, partial *η*^2^ = 0.23; 10,350–10,390: *F*_(2,46)_ = 13.43; *p* < 0.001, partial *η*^2^ = 0.37; 15,132–15,172: *F*_(2,46)_ = 4.73; *p* = 0.014, partial *η*^2^ = 0.17). Results of further paired comparisons showed that, for the time window of 5,532–5,572 ms, larger negativities were elicited by phrase violations than in the other two conditions (well-formed structures, *p* = 0.001; period violations, *p* = 0.024), whereas there was no significant difference in conditions between period violations and well-formed structures (*p* = 0.437). For the time window of 10,350–10,390 ms, larger negativities were elicited by phrase and period violations than in well-formed structures (phrase violations, *p* = 0.032; period violations, *p* < 0.001), while larger negativities were elicited by period violations than phrase violations (*p* = 0.047). For the time window of 15,132–15,172 ms, larger negativities were elicited by phrase violations than in the two other conditions (well-formed structures, *p* = 0.026; period violations, *p* = 0.036), whereas there was no significant difference in conditions between well-formed structures and period violations (*p* = 1.00).

### Time-Frequency Results

**Figure 4:**
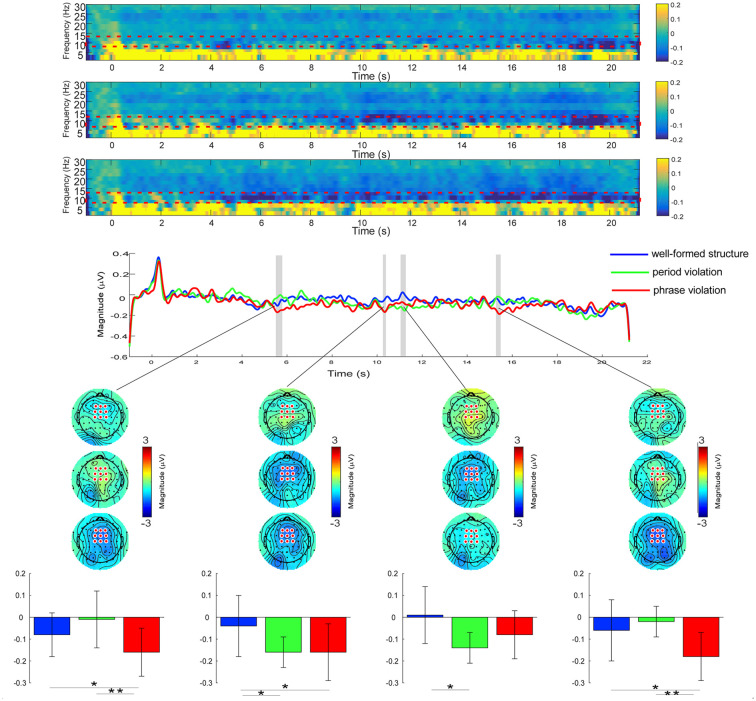
*Top*: the time-frequency analysis of electroencephalographic series in the three conditions at the FCz electrode. Alpha band frequencies (8–13 Hz) were selected as the regions of interest (ROI). The color scale represents the average decrease or increase of oscillation power. *Middle*: the alpha power at frontal-central electrodes during the whole music sequences. Significant regions are marked in the gray bar (*p* < 0.05, one-way ANOVA test, and FDR-corrected). *Bottom*: the scalp topography of the alpha band power for the three conditions within the significant time-window of 5,536–5,808 ms (736–1,008 ms after the onset of the second phrase), 10,284–10,432 ms (684–832 ms after the onset of the third phrase), 11,100–11,332 ms (1,500–1,732 ms after the onset of the third phrase), and 15,300–15,532 ms (900–1,132 ms after the onset of the last phrase). Colored bars represent the mean of alpha power in the three conditions for each time window (expressed as mean ± SD). *p < 0.05; **p < 0.05.

Figure 4 shows the time-frequency responses under different conditions. Significant time windows under different conditions for alpha frequency band (8–13 Hz) power are represented by gray-shaded areas. Scalp topographies at significant instants under different conditions reveal that the decrease in alpha power induced by phrase violations occurred around 800 ms after the onset of phrases 2–4, while this decrease lasted longer to period violations.

Results of a one-way RM ANOVA revealed significant main effects in the time windows of 5,536–5,808 ms (736–1,008 ms after the onset of the second phrase), 10,284–10,432 ms (684–832 ms after the onset of the third phrase), 11,100–11,332 ms (1,500–1,732 ms after the onset of the third phrase), and 15,300–15,532 ms (900–1,132 ms after the onset of the last phrase). For the time window of 5,536–5,808 ms, further paired comparisons indicated that the alpha band power induced by phrase violations was smaller than in the other two conditions (well-formed structures, *p* = 0.048; period violations, *p* = 0.007), whereas there was no significant difference in conditions between well-formed structures and period violations (*p* = 0.29). For the time window of 10,284–10,432 ms, smaller alpha band power was induced by phrase and period violations than well-formed structures (phrase violations, *p* = 0.038; period violations, *p* = 0.034), whereas there was no significant difference in conditions between phrase violations and period violations (*p* = 0.93). For the time window of 11,100–11,332 ms, smaller alpha band power was induced by period violations than well-formed structures (*p* = 0.014), whereas there was no significant difference between other comparisons (*p*s > 0.06). For the time window of 15,300–15,532 ms, smaller power was induced by phrase violations than in the other two conditions (well-formed structures, *p* = 0.050; period violations, *p* = 0.002), whereas there was no significant difference in conditions between well-formed structures and period violations (*p* = 0.51).

## Discussion

The current experiment examined the effects of long-range hierarchical structure on tension experience and its underlying neurocognitive mechanisms. The behavioral data showed that the violations of hierarchical structure induced high tension experience, which was not completely resolved at structural boundaries but accumulated during subsequent musical passages unfolding. The EEG results showed larger frontal-distributed negativities (N5) at GFP peaks, and decreasing power in the alpha band (8–13 Hz) in response to the structural violations. Compared to phrase violations, period violations elicited larger N5 and induced a longer-lasting decrease of power in the alpha band, indicating a hierarchical manner of musical processing. These results are discussed below in more detail.

### Dynamic and Accumulating Tension Experience as Music Unfolding

The behavioral tension ratings demonstrated that the tension induced by music is rarely static, but changing constantly over time, in line with a dynamic view of music emotion (Scherer and Moors, 2019). Previous studies investigating music emotion were mainly focused on a limited set of discrete emotion categories (e.g., pleasant vs. unpleasant) to uncover the specific relationship between some musical features and induced emotion (Menon and Levitin, 2005; Koelsch et al., 2006, 2013; Omigie et al., 2014). The methodology used in these studies cannot show the dynamic processes of musical emotion, therefore is inadequate to characterize the richness of emotion as the music unfolds over time. Our study was concerned with the dynamic process of musical tension as music unfolding in attempting to explore the development of musical tension and resolution.

Three tension curves showed a common trend across experimental conditions, in that musical tension always decreases towards the end of each phrase. The results suggested that musical boundaries were correlated with the resolution, resulting from the dominant or tonic chords in each phrase ending. Based on the relationship between the stability of chords and the strength of the generated tension (Bigand et al., 1996; Steinbeis et al., 2006), the harmonic cadence represented a strong conclusiveness, inducing the sense of resolution (Meyer, 1956; Lerdahl and Jackendoff, 1983).

A significant tension difference across conditions was found in the last three phrases. High tension experience was induced by structural violations, which can be explained by the Gestalt principles. Many aspects of music progression abide by Gestalt laws (Narmour, 1992). The sequences with phrase and period violations did not conform to the Gestalt principle of closure because of the tonality inconsistency. The violation of the Gestalt principle unfulfilled the prediction based on musical context, and in turn induced higher tension experience. In our study, the experimental design was careful and able to disentangle the influence of acoustic features and hierarchical structure on the experience of tension. In the first phrase, there was no significant difference in tension experience across conditions, although their acoustic features were different. In contrast, in the second phrase, the tension experience differed between sequences with well-formed structure and phrase violations, despite they had the same acoustic features.

The experience of tension was descendent but neither resolved completely nor immediately towards the end of each phrase but rather accumulated throughout subsequent musical pieces. In sequences with phrase violations, the tension induced by each incongruous tonality accumulated and resulted in higher starting points for the subsequent phrases. A similar situation was found in sequences with period violations. Our previous study investigating the tension experience induced by the nested structures also found the characteristics of tension experience accumulation (Sun et al., 2020a). The results could be explained by the event segmentation theory, which proposes a predictive error-based updating mechanism during the process of event segmentation (Zacks et al., 2007; Kurby and Zacks, 2008). When an unpredicted change occurs, event models are not maintained stable, but rather are updated based on current perceptual information. In our study, structural violations lead to predictive errors and event model resetting, which dynamically engaged working memory and cognitive control processes such as attention switching. This well explains why listeners’ felt tension increased at phrase boundaries.

It is worth noting that different patterns of tension and resolution induced by hierarchical structures in the present study and nested structures in the previous study (Sun et al., 2020a). The tension curves showed that more tension arches were induced in hierarchical structures than nested structures, which might due to the stronger dependence of tonality and more close connection in nested structures. However, it also might because only one musical phrase was included in sequences used by our previous study (Sun et al., 2020a), whereas four phrases in the present study, implying the important role of the organization of musical units on tension experience. Future studies should further investigate the difference of tension experience induced by nested structures and hierarchical structures to discriminate tension experience delicately and shed new light on structural processing.

### The Cognitive Neural Mechanism Underlying the Tension Experience

Given our experiment was the first study to investigate the dynamic EEG underlying musical tension experience, we adopted a data-driven method to determine the significant time windows. The topographical approach of GFP does not require any* a priori* hypothesis, such as the prerequisite of electrodes and time intervals, and therefore is amenable to determining significant time points statistically and reliably. GFP in EEG signal reflects stronger global brain activity, its value represents the strength of the electric potential over all EEG electrodes on the scalp, and has been used in previous studies to measure the global brain activity (Hu et al., 2014; Khanna et al., 2015). In our study, to assess the dynamic process evoked by the whole musical pieces, we analyzed epochs as long as 21.2 s. It would be inadequate to use traditional ERP analytical methods with low high pass filters because it was hard to exclude slow-wave drifts due to the long time-span of the musical pieces. GFP is superior in eliminating the influence of a poor signal-to-noise ratio (Milz et al., 2017), therefore suitable for characterizing rapid changes in brain activity.

It was found that frontal-distributed negativities at GFP peaks always occurred around 600–800 ms after the structural violations, the latency and scalp distributions were similar to the N5 component. Previous studies have suggested that the N5 is related to the violation of expectation and the integration of incongruent harmonic chord into musical context (Koelsch, 2005; Miranda and Ullman, 2007; Steinbeis and Koelsch, 2008; Koelsch and Jentschke, 2010). In the time-frequency domain, structural violation induced a decrease in central-frontal alpha power. The decreased power in the alpha band may reflect orienting responses (Klimesch, 1999; Krause, 2006) and attention switching (Meyer et al., 2013; Sadaghiani and Kleinschmidt, 2016). In our experiment, listeners constructed a psychological model to predict the upcoming musical events based on musical contexts. Musical violations led to prediction errors, which required the brain to change the comparatively stable model to reduce prediction errors for the upcoming events. Event model resetting engaged more working memory and attention, the neural bases of which were reflected in the decreased power in the alpha band. Furthermore, more cognitive resources were devoted to the integration of harmonic structures, as indicated by the larger N5 component.

It is worth noting that the N5 effects elicited by period violations were larger than phrase violations because it is more difficult for listeners to integrate the period violations into the musical context (Zhang et al., 2018). Furthermore, longer-lasting decreases in alpha power were induced by period violations (around 800 ms to 1,500 ms) compared with the phrase violations (around 800 ms), suggesting more attentional resources were paid for the high-level violations. The results suggested a hierarchical manner of music processing. In line with previous studies in both music and language domains (Ding et al., 2016; Zhang et al., 2016; Harding et al., 2019), our study indicated that structural processing at different hierarchical levels might engage different neural mechanisms. Furthermore, the results supported the predictive coding theory (Friston, 2005, 2010), pointing to the hierarchical predictive coding process inherent to the auditory system, and active construction while listening to music (Koelsch, 2018).

### The Relationship Between Tension Ratings and EEG Brain Responses

Compared with well-formed sequences, larger N5 and decreased power in the alpha band were shown when listening to the music sequences with structural violations at phrase and period levels. The effects of these distinct brain responses were in parallel with the changes of behavioral tension ratings, in that the close corresponding relationship is indicated by the similar time windows. The modulation of musical structure on tension ratings and brain responses can be explained by the predictive processes. The mismatch between the actual stimulus and the prediction induced tension experience (Meyer, 1956; Margulis, 2005; Huron, 2006), which required listeners to pay more cognitive resources to update working memory, switch attention (Zacks et al., 2007; Kurby and Zacks, 2008) and integrate the violated events into a musical context, as implied by large N5 and decrease power in the alpha band.

Although tension ratings and brain responses induced by music structural violations are closely related, the processing processes they reflect are different. In particular, tension ratings reflected the online tension experience, while the brain responds to the integrated processing of the musical event into the musical context. Thus, the tension rating induced by each structural violation was based on the ending point in the previous phrase. That is, the tension value in one moment represented the tension induced by the present musical event and the accumulated tension from the previous musical context. In contrast, the N5 and the power in the alpha band did not exhibit the accumulation characteristics, but fading away within one phrase. The strength of the brain responses only reflected the cognitive effort to integrate the present musical event into the musical context.

In conclusion, the present study using behavioral rating and EEG investigated the influence of music hierarchical structure on the experience of tension and its underlying neural mechanism. Behavioral results showed that structural violations gave rise to an increasing and accumulating tension experience as indicated by tension curves. In parallel, brain responses including larger N5 effects at GFP peaks and decreased power in alpha frequency band were in response to the structural violations, reflecting more attention and cognitive resources engaged in the integrated processing. Moreover, compared with phrase violations, period violations elicited larger N5 and induced a longer-lasting decrease in alpha power, revealing the hierarchical manner of structure processing in music. The dynamic tension experience revealed by our findings is of significance to emotional regulation. Furthermore, the hierarchical manner of structure processing also reminded musicians to pay more attention to large-scale music structure while listening to music.

## Data Availability Statement

The raw data supporting the conclusions of this article will be made available by the authors, without undue reservation.

## Ethics Statement

The studies involving human participants were reviewed and approved by Institutional Review Board of the Institute of Psychology, Chinese Academy of Sciences. The patients/participants provided their written informed consent to participate in this study.

## Author Contributions

LS and YY proposed the study and designed the experiment. LS and GR conducted the tests. LS and LH contributed to data analysis. All authors contributed to the article and approved the submitted version.

## Conflict of Interest

The authors declare that the research was conducted in the absence of any commercial or financial relationships that could be construed as a potential conflict of interest.
